# Gender-specific insights into *TTN* mutation: potential biomarker for female risk in kidney renal clear cell carcinoma

**DOI:** 10.1016/j.jgeb.2025.100506

**Published:** 2025-06-04

**Authors:** Ayan Saha, Senzuti Sharmin, Tazin Ahmed, Sadia Tabassum, Ayan Roy, Pallab Kar, Paromita Biswas, Jannatul Ferdoush

**Affiliations:** a Department of Biological Sciences, Asian University for Women, Chittagong 4000, Bangladesh; b Department of Biochemistry and Microbiology, North South University, Dhaka 1229, Bangladesh; c Science and Math Program, Asian University for Women, Chattogram 4000, Bangladesh; d Department of Applied Science, Wrexham Glyndwr University, Wrexham LL11 2AW, United Kingdom; e Department of Genetic Engineering and Biotechnology, East West University, Dhaka 1212, Bangladesh; f African Medicinal Flora and Fauna Research Niche, Walter Sisulu University, Mthatha 5117, South Africa; g Department of Biology, Geology and Environmental Science, University of Tennessee at Chattanooga, 615 McCallie Ave, Chattanooga, TN 37403, USA

**Keywords:** KIRC, TTN, Prolactin signaling pathway, Gender differences, ccRCC

## Abstract

Kidney Renal Clear Cell Carcinoma (KIRC) is a leading cause of cancer death worldwide, but its early detection remains hindered by a lack of genetic markers. Our study aims to find prospective biomarkers that could serve as prognostic indicators and help in the identification of efficient drug candidates for KIRC treatment. Importantly, this study identifies the hub genes that play a crucial role in KIRC and their impact on male and female patients. The cBioPortal was used to identify frequently mutated genes across seven KIRC studies. Additionally, GSE168845 was employed to identify the differentially expressed genes. The analysis revealed that the titin (TTN) gene was mutated and upregulated in KIRC. Subsequently, differential genes of wild-type TTN versus mutant TTN were identified using TNMplot. The NetworkAnalyst tool was used to conduct KEGG analysis and PPI analysis on these genes. Furthermore, the Kaplan-Meier Plotter was utilized to perform overall survival analysis. Our findings indicated that the TTN gene leads to poorer prognosis in women than in men. We also discovered that the female-specific prolactin signaling pathway plays a significant role in the progression of KIRC. Moreover, our study suggested that the GDF15 gene, involved in the prolactin signaling pathway, has a worse prognosis for KIRC in women than in men. Additionally, mRNA expression analysis showed a negative correlation between GDF15 and MAPK14 in KIRC. Collectively, our research indicates that TTN, GDF15, and MAPK14 can serve as prognostic biomarkers in female KIRC patients, offering prospects for enhanced treatment and patient outcomes in these cancers.

## 1 Introduction

Kidney renal clear cell carcinoma (KIRC), a predominant subtype of renal cell carcinoma (RCC), represents 75% of all RCC cases.^1^ RCC is the most prevalent and devastating kidney malignancy in adults, which affects the human genitourinary system.^2^ There are several types of RCC including KIRC and the recovery rate of KIRC is lower than the other types of RCC.^3^ Notably, KIRC does not exhibit a progressive response to radiotherapy or chemotherapy treatment, leading to a high mortality rate.^4^ Since KIRC shows resistance to radiotherapy and chemotherapy treatment, surgical removal is the only remaining effective treatment for RCC.^5^ While immunotherapy and targeted therapy options are available for RCC treatment, the prognosis remains poor for the patients who are incompetent of surgical treatment.^6^ Therefore, a comprehensive treatment approach that contains surgical interventions and targeted therapies has been used for managing KIRC, resulting in significant advancements in enhancing patient outcomes.^7^

KIRC is characterized by the accumulation of clear cells within the kidney, derived from the proximal tubules and exhibiting distinctive metabolic and genetic features. Gene mutations have been implicated in KIRC pathogenesis and progression. For example, mutations in the *VHL* gene have been found in approximately 70–80 % of KIRC cases,^8^ resulting in the disruption of HIF1α and HIF2α, which control the expression of several genes related to metabolism and chromatin remodelling and angiogenesis.^9^ In addition, several other genes, including *PBRM1*, *BAP1*, *SETD2*, *KDM6A*, *KDM5C*, *and MLL2* are frequently mutated or deleted in KIRC, suggesting their potential role in KIRC pathogenesis.^10^

Furthermore, gender-specific differences, which are not fully understood but may be related to differences in hormone levels or genetic susceptibility, might play a role in KIRC progression. Several studies have demonstrated the presence of gender-specific disparities in renal disorders. For example, women are more likely than men to have chronic kidney disease (CKD),[11], [12] However, in 2015, the United States Renal Data System (USRDS) reported that 62% of patients with CKD who progressed to end-stage renal failure were male, whereas only 38% were female.^13^ Moreover, men have a twofold higher likelihood of developing kidney cancer compared to women and also experience a higher mortality rate.^14^ Men have been found to have a higher incidence of RCC than women, with a male-to-female ratio of approximately 2:1, and male RCC patients often have larger tumors and a worse prognosis compared to female RCC patients.^15^

In summary, KIRC is a complex disease with multiple genetic and epigenetic alterations contributing to disease progression. Gender-specific differences in disease progression have also been observed, highlighting the importance of personalized treatment strategies. Hence, this study aims to investigate the key genes and molecular mechanisms involved in KIRC and determine their prognostic significance, with a specific focus on gender differences. It seeks to understand how genetic variations impact the prognosis of KIRC in men and women and to identify potential gender-specific therapeutic targets.

## 2 Methodology

### 2.1 Data sources

#### 2.1.1 Patient cohort analysis in cBioPortal

**Fig. 1:**
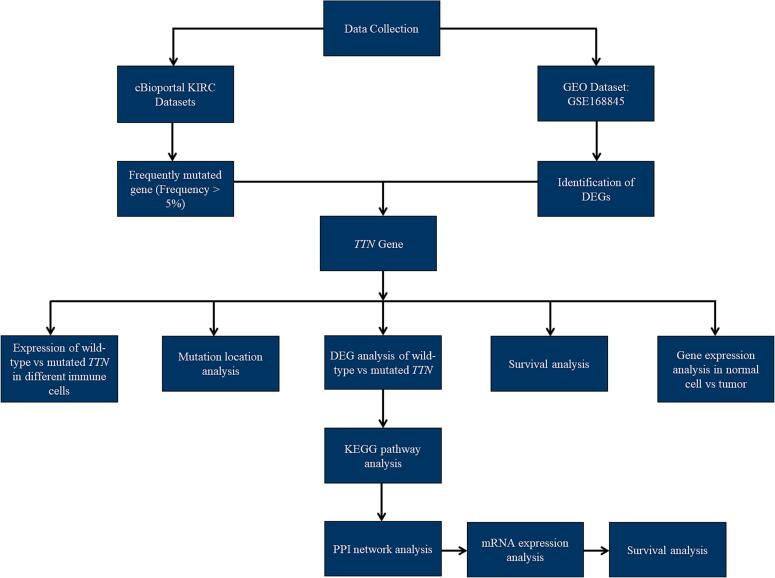
Overview of the study workflow for *TTN* gene analysis in kidney renal clear cell carcinoma (KIRC): The workflow begins with data collection from two sources: the cBioPortal KIRC datasets (for identifying frequently mutated genes) and the GEO dataset (GSE168845) for differential gene expression (DEG) analysis. The *TTN* gene is selected for further investigation, including mutation location analysis and gene expression comparisons between wild-type and mutant *TTN* in immune cells. Additional analyses include DEG analysis between wild-type and mutant *TTN* samples, survival analysis, and gene expression comparisons between normal and tumor tissues. Functional enrichment analyses, such as KEGG pathway analysis and protein–protein interaction (PPI) network analysis, are then performed, followed by mRNA expression and survival analyses. (Fig. 1).

The present study utilized cBioPortal to select seven KIRC datasets, including TCGA Firehose Legacy, TCGA Nature 2013, TCGA PanCancer Atlas, DFCI Science 2019, BGI Nat Genet 2012, IRC Nat Genet 2014, and UTokyo Nat Genet 2013, to identify the most frequent mutations. Furthermore, the study investigated the survival rates of patients with *TTN* mutations compared to those with wild-type *TTN* across these seven studies. Additionally, the location of *TTN* mutations within the protein was also examined using cBioPortal. Lastly, Kaplan-Meier survival analysis was conducted for male and female KIRC patients with *TTN* mutations (Fig. 1).

#### 2.1.2 Differential gene expression analysis in KIRC patients using the Gene Expression Omnibus (GEO) database

The microarray dataset with accession number “GSE168845” was downloaded from the GEO database.^16^ The dataset consisted of four KIRC tissues and paired non-cancerous renal tissue (NRT) samples. Only the cancer and normal kidney tissue samples were used for analysis (Fig. 1). The online tool GEO2R (RRID:SCR_016569, https://www.ncbi.nlm.nih.gov/geo/geo2r/) was used to identify differentially expressed genes (DEGs) by comparing KIRC vs NRT. The Benjamini-Hochberg false discovery method was used to adjust p-values. DEGs were considered significant if |logFC| ≥ 1.5 and the adjusted p-value (adj.p) was <0.05.

### 2.2 *TTN* expression in normal and KIRC tissue samples

TNMplot (https://tnmplot.com/analysis/), a web-based tool for visualizing and comparing gene expression data across normal, tumor, and metastatic tissues,^17^ was used to analyse *TTN* gene expression between normal and KIRC tissues (Fig. 1). The Mann–Whitney *U* test was applied to compare *TTN* expression levels between KIRC tumor and normal kidney tissues.

### 2.3 TIMER analysis of the *TTN* gene in KIRC

The Tumor Immune Estimation Resource (TIMER, https://cistrome.shinyapps.io/timer) database enables in-depth analysis of the molecular characteristics of tumor–immune interactions (Fig. 1). This study utilized TIMER to assess the infiltration levels of various immune cell types, including B cells, CD8^+^ T cells, CD4^+^ T cells, macrophages, neutrophils, and dendritic cells, in KIRC patients with mutated and wild-type *TTN*. Statistical significance of differential infiltration was evaluated using the Wilcoxon test, with p-values < 0.05 considered indicative of significant differences.

### 2.4 muTarget analysis between wild-type and mutant *TTN* in KIRC

The Mutational Target Identification Portal, available at https://www.mutarget.com, was used to identify differentially expressed genes (DEGs) between mutant and wild-type *TTN* in KIRC (Fig. 1). RNA sequencing and mutation data were obtained from TCGA. Data processing was performed in the R environment using the DESeq2 algorithm for normalization, and transcript variants were annotated using the AnnotationDbi. Differential expression analysis was conducted between TTN-mutant and wild-type samples using the Mann-Whitney *U* test.

### 2.5 Protein–protein interaction (PPI) network construction and KEGG pathway enrichment

To investigate the functional relationships between genes with differential expression patterns in *TTN-*mutated and wild-type KIRC samples, protein–protein interaction networks (PPI) were constructed using the Search Tool for the Retrieval of Interacting Genes/Proteins (IMEx Interactome)^18^ and the NetworkAnalyst online platform.^19^ Additionally, the KEGG pathway was enriched (with an adjusted p-value of less than 0.05) through the NetworkAnalyst platform (Fig. 1).^19^

### 2.6 Kaplan-Meier survival analysis

Gender-specific survival analysis of the *GDF15* and *MAPK14* genes was performed using Kaplan-Meier Plotter (https://www.kmplot.com/analysis/) (Fig. 1). It is an online tool for survival analysis.^20^ The mRNA RNAseq option and auto select best cutoff options were selected for the overall survival analysis.

## 3 Result

### 3.1 *TTN* gene mutation as a potential risk factor in female patients with KIRC

**Fig. 2:**
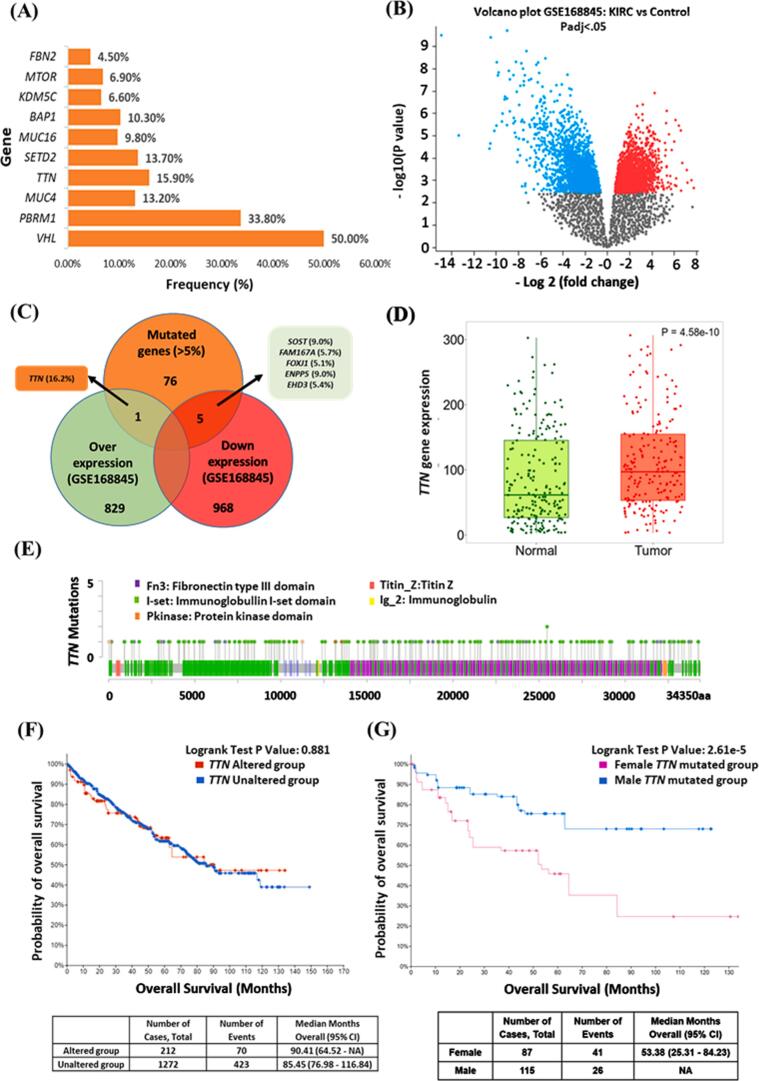
Genomic alterations, expression patterns, and survival outcomes in KIRC patients. (A) Frequently mutated genes in KIRC, (B) Differentially expressed genes (DEGs) identified from the GSE168845 dataset, (C) Venn diagram of mutated genes and DEGs, (D) *TTN* gene expression in normal tissue and tumor tissue, (E) Mutation locations within the *TTN* gene across KIRC samples, (F) Survival analysis comparing patients with wild-type and mutated *TTN* and (G) Survival analysis of men and women KIRC patient with mutated *TTN*.

The cBioPortal analysis identified the most frequently mutated genes and their corresponding mutation frequencies in KIRC (Fig. 2A). The results showed that the most frequently mutated genes included *VHL* (50 %), *PBRM1* (33.8 %), *MUC4* (13.2 %), *TTN* (15.9 %), *SETD2* (13.7 %), *MUC16* (9.8 %), *BAP1* (10.3 %), *KDM5C* (6.6 %), *MTOR* (6.9 %), and *FBN2* (4.5 %). Later, using the GEO2R tool, 829 upregulated and 968 downregulated DEGs were identified in the GSE168845 microarray dataset. The result was presented in a volcano plot (Fig. 2B) where red demonstrated upregulated genes, and blue indicated downregulated genes. Subsequently, the Venn diagram (Fig. 2C) suggested that only one gene was common in over-expressed DEGs and mutated genes (mutation >5 %), which was the *TTN* gene. On the other hand, five genes—*SOST*, *FAM167A*, *FOXJ1*, *ENPP5* and *EHD3*— were common between downregulated DEGs and frequently mutated genes (mutation >5 %). Gene expression analysis showed that TTN expression was higher in tumor tissue than in normal tissue (Fig. 2D). Furthermore, the muTarget result identified mutations in five domains of the *TTN* gene in KIRC. These domains included the fibronectin type III (fn3), immunoglobulin I-set (I-set), protein kinase (Pkinase), titin 2 (Titin_2), and immunoglobulin domain (Ig_2) (Fig. 2E). Notably, mutations were distributed accross all five areas. The survival analysis showed no significant difference in the survival rates of individuals with wild-type *TTN* and mutant *TTN* (Fig. 2G) in KIRC patients. Surprisingly, female individuals with the *TTN* mutations had a worse prognosis than male individuals (Fig. 2F).

### 3.2 *TTN* mutation reduces the level of immune cell infiltration

**Fig. 3:**
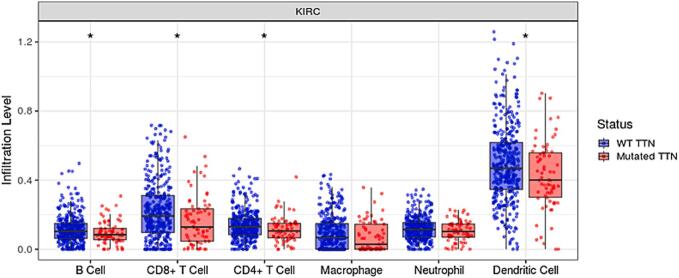
Infiltration levels of different immune cells in wild-type *TTN* and mutated *TTN* KIRC patients.

In KIRC patients with mutated *TTN*, the infiltration levels of B cells, CD8^+^ T cells, CD4^+^ T cells, and dendritic cells were significantly lower compared to those with wild-type *TTN* (Fig. 3). Due to mutations in the *TTN* gene, the immune response may be reduced in KIRC patients.

### 3.3 Pathway analysis of differentially expressed genes (DEGs) in *TTN*-mutated KIRC

**Fig. 4:**
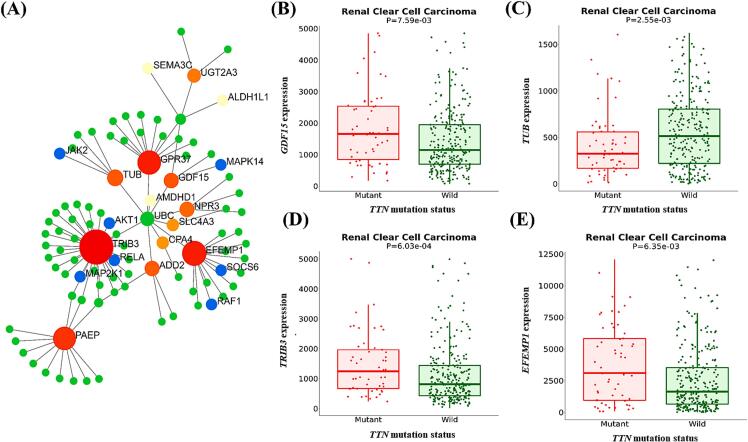
Interaction and associations of DEGs in *TTN-*mutated KIRC patients. (A) PPI network of DEGs between wild-type and *TTN* mutated KIRC tissue where blue indicates the proteins associated with Prolactin signaling pathway. The expression levels of (B) *GDF15*, (C) *TUB*, (D) *TRIB3*, and (E) *EFEMP1* gene in wild-type *TTN* and mutated *TTN* were also analysed. These genes are interact with DEGs associated with the prolactin signaling pathway. (For interpretation of the references to colour in this figure legend, the reader is referred to the web version of this article.)

KEGG pathway analysis revealed that the DEGs were significantly associated with different pathways, for instance, “signaling pathways regulating pluripotency of stem cells”, “protein processing in endoplasmic reticulum”, “T cell receptor signaling pathway”, “neurotrophin signaling pathway”, “hepatitis B”, “Fc epsilon RI signaling pathway”, “AGE-RAGE signaling pathway in diabetic complications”, “prolactin signaling pathway”, and “chronic myeloid leukemia” (Table 2). Due to the pronounced effects of gender-specific hormones on the prolactin signaling pathway, this study will further focus on genes associated with this pathway that are responsive to hormonal regulation. In the prolactin signaling pathway, the proteins SOCS6, RAF1, MAP2K1, AKT1, JAK2, RELA, and MAPK14 proteins were found to directly interact with DEGs (Table 1) in *TTN*-mutated KIRC patients (Fig. 4A). Among the DEGs, while the expression levels of *GDF15* (Fig. 4B), *TRIB3* (Fig. 4D) and *EFEMP1* (Fig. 4E) expression was higher in *TTN*-mutated tissue, the expression of *TUB* (Fig. 4C) was lower in mutated type *TTN*. The interactions between proteins encoded by these DEGs and proteins of the prolactin signaling pathway were further assessed by evaluating mRNA co-expression in KIRC patients using cBioPortal.

**Table 1:** Upregulated and Downregulated genes in *TTN* mutated KIRC patients.

Gene	Mean *TTN* mutant (n = 58)	Mean *TTN* wild (n = 274)	FC (mutant/wild)	Expression	p-value
*ALDH1L1*	1990.67	2939.26	1.47	Down	2.16E−03
*AMDHD1*	93.74	138.38	1.47	Down	7.92E−03
*NPR3*	4469.09	7856.01	1.75	Down	4.56E−03
*PAEP*	38.78	398.02	10	Down	6.67E−03
*RUNDC3B*	100.14	145.46	1.45	Down	9.72E−03
*TRHDE-AS1*	168.1	245.82	1.47	Down	2.70E−03
*TUB*	414.45	607.11	1.47	Down	2.55E−03
*UGT2A3*	2931.57	4429.02	1.52	Down	7.01E−03
*ADD2*	184.28	97.27	1.89	Up	4.95E−03
*C14orf132*	420.48	269.41	1.56	Up	6.87E−03
*CPA4*	434.64	118.27	3.67	Up	3.82E−04
*EFEMP1*	4201.59	2888.96	1.45	Up	6.35E−03
*FMOD*	1294.64	855.3	1.51	Up	7.62E−03
*GDF15*	2521.28	1753.02	1.44	Up	7.59E−03
*GPR37*	183.19	119.34	1.54	Up	7.39E−04
*LIF*	1188.22	813.16	1.46	Up	6.20E−03
*NEFL*	3053.86	1320.27	2.31	Up	1.61E−03
*SEMA3C*	1540.91	1037.19	1.49	Up	4.18E−03
*SLC4A3*	176.24	101.1	1.74	Up	4.85E−03
*TRIB3*	2062.76	1326.56	1.55	Up	6.03E−04
*TRNP1*	206.28	113.8	1.81	Up	4.60E−03

*FC = Fold Change.

**Table 2:** KEGG pathway analysis of the DEGs.

Pathway	Total	Expected	Hits	p-value	FDR	Genes
Signaling pathways regulating pluripotency of stem cells	139	1.17	11	1.65E−08	5.26E−06	*RIF1, SMAD3, RAF1, ONECUT1, MAP2K1, AKT1. JAK2, HNF1, ABMPR2, SETDB1, MAPK14*
Protein processing in endoplasmic reticulum	165	1.39	11	9.83E−08	1.42E−05	*STUB1, DNAJB1, PDIA3, ATF4, MAP2K7, PRKNHSPA1, BHSPA8, DNAJA1, DDIT3, SYVN1*
T cell receptor signaling pathway	101	0.849	9	1.34E−07	1.42E−05	*RAF1, MAP2K1, MAP2K7, AKT1, FYN, PTPR, PLCG1, RELA, MAPK14*
Neurotrophin signaling pathway	119	1	9	5.51E−07	4.38E−05	*RAF1, MAP2K1, ATF4, MAP2K7, AKT1, ABL1, PLCG1, RELA, MAPK14*
Hepatitis B	163	1.37	10	8.63E−07	5.49E−05	*SMAD3, RAF1, MAP2K1, ATF4, MAP2K7, AKT1, JAK2, ARAF, RELA, MAPK14*
Fc epsilon RI signaling pathway	68	0.571	7	1.38E−06	6.67E−05	*RAF1, MAP2K1, MAP2K7, AKT1, FYN, PLCG1, MAPK14*
AGE-RAGE signaling pathway in diabetic complications	100	0.84	8	1.61E−06	6.67E−05	*SMAD3, NOS3, AKT1, JAK2. FN1, PLCG1, RELA, MAPK14*
Prolactin signaling pathway	70	0.588	7	1.68E−06	6.67E−05	*SOCS6, RAF1, MAP2K1, AKT1, JAK2, RELA, MAPK14*
Chronic myeloid leukemia	76	0.638	7	2.94E−06	0.000104	*SMAD3, RAF1, MAP2K1, AKT1, ABL1, ARAF, RELA*

*FDR = False Discovery Rate.

### 3.4 Exploring the relationship between DEGs in *TTN*-mutated KIRC and prolactin signaling pathway components

**Fig. 5:**
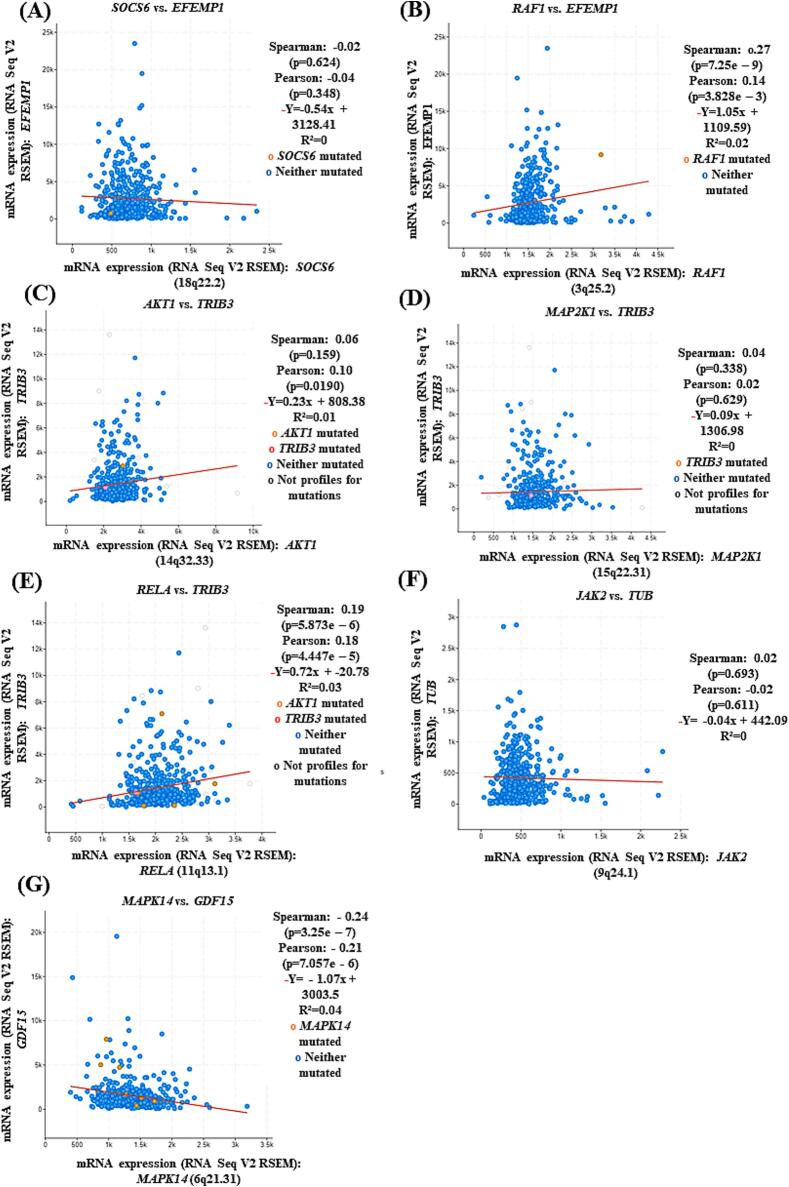
Spearman and Pearson correlations of mRNA expression in KIRC patients were assessed between directly interacting proteins involved in the prolactin signaling pathway. The correlations analyzed include (A) *SOCS6* vs. *EFEMP1*, (B) *RAF1* vs. *EFEMP1*, (C) *AKT1* vs. *TRIB3*, (D) *MAP2K1* vs. *TRIB3*, (E) *RELA* vs. *TRIB3*, (F) *JAK2* vs. *TUB*, and (G) *GDF15* vs. *MAPK14*.

The mRNA analysis showed that *RAF1* and *EFEMP1* (Fig. 5B), *AKT1* and *TRIB3* (Fig. 5C), and *RELA* and *TRIB3* (Fig. 5E) were positively and significantly correlated in KIRC patients. In contrast, *SOCS6* and *EFEMP1* (Fig. 5A), as well as *GDF15* and *MAPK14* (Fig. 5G), were significantly negatively correlated. No significant correlation was observed between *MAP2K1* and *TRIB3* (Fig. 5D) or *JAK2* and *TUB* (Fig. 5F). This study further analysed the risk associated with DEGs that are significantly correlated with genes in the prolactin signaling pathway.

### 3.5 Gender-specific survival analysis of genes associated with the prolactin signaling pathway

**Fig. 6:**
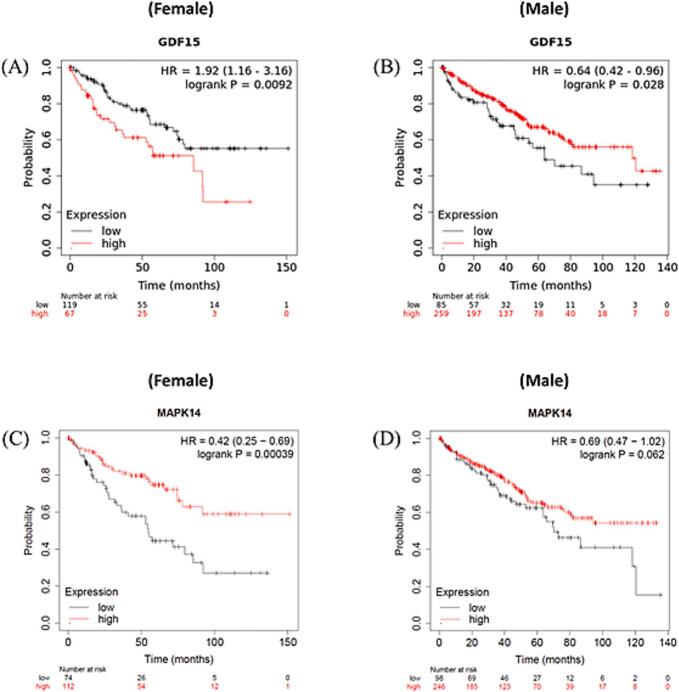
Survival analysis was performed based on the expression of GDF15 in (A) female and (B) male, and MAPK14 in (C) female and (D) male KIRC patients.

The survival analysis revealed that females with higher *GDF15* expression had a faster mortality rate than those with lower expression levels (Fig. 6A). Conversely, males with lower *GDF15* expression levels showed a more rapid mortality rate compared to those with higher expression levels (Fig. 6B). Lower expression of *MAPK14* was associated with significantly decreased survival rates in male patients, while it was linked to increased, though not statistically significant, survival in female patients (Fig. 6C and D). From the correlation analysis and gender-specific survival analysis, it is evident that *GDF15* and *MAPK14* are negatively correlated in male and female KIRC patients.

## 4 Discussion

The *TTN* gene encodes titin, the largest protein in the human body, which is a crucial part of the sarcomere.^21^ In this study, we showed that overexpression and misexpression of the *TTN* gene are associated with KIRC. Notably, the *TTN* gene has been shown to be frequently mutated in KIRC. An study reported that mutations in the *TTN* gene indicate a high tumor mutation burden (TMB) state in 33 cancer types, including KIRC.^22^ To analyse mutant genes, including *TTN*, a group of researchers created a KIRC evolutionary path using Bayesian Mutation Landscape.^23^ They found that although *TTN* was at the base of the evolutionary tree, it had a low out-degree and fewer subsequent mutations. This observation suggested that *TTN* plays a role as a passenger gene in KIRC. Furthermore, another study indicated that *TTN* was one of the top five most frequently mutated genes in low-expression and high-expression subtype CD8^+^ T cell-associated genes in KIRC, with 20.8% and 24.4% mutation frequency, respectively.^24^ It is important to note that CD8^+^ T cells can precisely identify and eliminate cancerous cells.^25^ Hence, mutation in the *TTN* gene in KIRC patients might affect CD8^+^ cells and hinder their ability to eradicate cancerous cells. Additionally, one study found that TTN antisense RNA 1 is excessively expressed in KIRC and functions as an oncogenic lncRNA by acting as a ceRNA for miR-195.^26^ It also positively controls the production of cyclin D1 in KIRC cells. Thus, the *TTN* gene has been reported to be overexpressed, misexpressed, or mutated frequently in several cancers, including KIRC. However, it is not clearly understood how the *TTN* gene is commonly mutated in KIRC. Therefore, it is critical to understand the molecular mechanism to elucidate the relationship between *TTN* and KIRC in order to develop future potential therapeutics.

To understand it, we first performed KEGG pathway analysis on the DEGs of mutated *TTN* and wild-type *TTN* KIRC patients and found that the prolactin signaling pathway is linked to KIRC. The prolactin signaling pathway is activated when prolactin, a sex hormone^27^ that is produced and released by specialized cells called lactotrophs in the anterior pituitary gland^28^ binds to the prolactin receptor.^29^ Prolactin plays essential roles in initiating and maintaining lactation, facilitating pregnancy implantation, promoting the growth and specialization of mammary gland cells, regulating the immune system, and stimulating the formation of new blood vessels.^28^ While both males and females possess the prolactin hormone, breastfeeding women exhibit elevated levels of prolactin, and when the breast is stimulated, prolactin levels in lactating women increase significantly, nearly doubling the baseline level.^30^ Moreover, prolactin secretion is mainly regulated by estradiol, which is the major sex hormone in female,^31^ and fluctuations in prolactin levels were seen throughout the menstrual cycle, with the peak occurring either during ovulation or the luteal phase.^32^ Thus, our study uncovered a significant connection between the *TTN* gene, KIRC, and the prolactin signaling pathway, suggesting that the female-specific prolactin signaling pathway plays a significant role in the progression of KIRC.

**Fig. 7:**
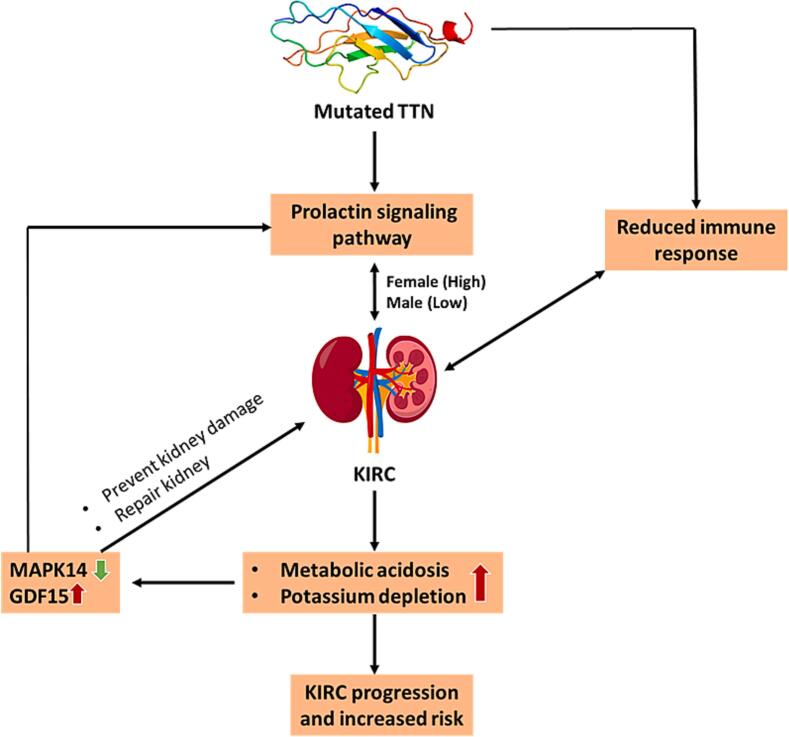
Possible mechanisms behind high risk of the *TTN* mutated female KIRC patient. Mutated TTN regulates the prolactin signaling pathway and reduces the immune response, promoting kidney renal clear cell carcinoma (KIRC). Prolactin signaling is more active in female patients than in male patients. The regulation of KIRC and prolactin signaling pathways is reciprocal, with KIRC further exacerbating metabolic acidosis, potassium depletion, GDF15 overexpression, and MAPK14 downregulation. GDF15 overexpression enhances kidney repair mechanisms but also dysregulates the prolactin signaling pathway, potentially increasing the risk and progression of KIRC, particularly in female patients.

Next, we wanted to see if *TTN* gene mutation has any correlation with other genes. To test it, we performed DEG analysis and found that mutation in the *TTN* gene resulted in upregulation of GDF15 expression in KIRC. Elevated expression of GDF15 protein in outer medullary collecting duct cells has been observed in cases of metabolic acidosis and potassium depletion, both of which are linked to decreased kidney function (Fig. 7).[33], [34] Another study also revealed that a rise in prolactin levels was linked to a decrease in kidney function from stages 1 to 5 of chronic kidney disease (CKD) in postmenopausal women.^35^ Research has shown that elevated levels of GDF15 have been associated with a higher likelihood of developing chronic kidney disease and a more rapid deterioration of kidney function in different types of renal disorders, such as diabetic nephropathy, IgA nephropathy, lupus nephritis, anti-glomerular basement membrane nephritis, primary membranous nephropathy, kidney transplantation, Fabry disease, and amyloidosis.^36^ Another study revealed that individuals diagnosed with renal cell carcinoma (RCC) exhibited elevated levels of serum GDF15 compared to the control group.^37^ Interestingly, there was a connection between GDF15 and gut microbiota-derived trimethylamine N-oxide (TMAO), specifically in females who were experiencing end-stage kidney disease.^38^ TMAO is a byproduct formed by oxidation by gut bacteria that break down lipids, including choline and compounds similar to carnitine.^39^ Different studies have suggested that TMAO is closely related to kidney diseases.[40], [41], [42], [43], [44] The presence of TMAO may stimulate the upregulation of GDF15, leading to the development of KIRC. However, another study showed that the proliferation of acid-secreting collecting duct cells relies on GDF15 expression, suggesting that GDF15 plays a role in maintaining tubular integrity and promoting repair.^45^ Intracranial GDF15 may also protect against organ damage by modulating inflammatory cell recruitment, as observed in myocardial infarction and early diabetic kidney disease models.^46^ Thus, overexpression of GDF15 can both positively and negatively regulate kidney disease.

Our study further showed that GDF15 is inversely correlated with MAPK14. One study found that downregulation of MAPK14 and P‐MAPK14 can hinder the proliferation and migration of clear cell renal cell carcinoma by reducing the expression of CDC25B.^47^ However, in *Villin-Cre; TSC1^f/f^* mice, the deletion of *MAPK14* further enlarged kidney size, increased cyst formation, and worsened renal secretion issues. TSC-associated RCCs are diverse in their characteristics, including a tendency to affect females more often, an earlier age of onset, and the presence of multiple tumors within the kidneys (multifocality).^48^ Therefore, further investigation is required to understand the mechanisms behind the downregulation of MAPK14 and its associated risks in female KIRC patients. Although there is currently no research on the association between GDF15 and MAPK14 in renal disease, a study has demonstrated the presence of the GDF15/MAPK14 axis in osteoarthritis.^49^ Future research could investigate the role of the GDF15/MAPK14 axis in KIRC. *In vitro* and *in vivo* investigations should be a part of future research to validate these findings and explore the function of the prolactin signaling pathway in female KIRC patients with *TTN* mutations. Furthermore, the comparatively small open-access dataset used in this study might not adequately represent the diversity of KIRC patients. However, our results provide an insightful foundation for understanding gender-specific processes in KIRC and present promising targets for diagnostics and treatment in patients with *TTN* mutations.

## 5 Conclusion

Our findings indicate that the presence of *TTN* mutation in women is associated with worse prognosis than in men. In addition, we have demonstrated that prolactin signaling pathway, which plays a significant role in female physiology is related to KIRC disease. Finally, prolactin pathway-associated genes *GDF15* and *MAPK14* might have a negative correlation and are linked to poor disease progression in female KIRC patients. GDF15 has been shown to activate the MAPK/ERK1/2 signaling cascade through increased ERK1/2 phosphorylation in various cell types, including cancer cells; although a direct connection to prolactin signaling has not been established, it is possible that this pathway is regulated indirectly or as a secondary response. These findings underscore the importance of considering gender-specific molecular mechanisms in cancer research. Targeting these pathways may lead to more effective, personalized therapies for *TTN*-mutated KIRC patients.

## CRediT authorship contribution statement

**Ayan Saha:** Writing – review & editing, Supervision, Project administration, Methodology, Funding acquisition, Data curation, Conceptualization. **Senzuti Sharmin:** Formal analysis. **Tazin Ahmed:** Visualization, Validation. **Sadia Tabassum:** Writing – original draft, Visualization. **Ayan Roy:** Conceptualization. **Pallab Kar:** Supervision, Software, Project administration. **Paromita Biswas:** Visualization. **Jannatul Ferdoush:** Writing – original draft, Supervision, Investigation, Conceptualization, Funding acquisition.

## Declaration of competing interest

The authors declare the following financial interests/personal relationships which may be considered as potential competing interests: This work is supported by the internal faculty grant of Ayan Saha at the Asian University for Women, Bangladesh, and the startup fund of Jannatul Ferdoush at the University of Tennessee at Chattanooga, USA.
